# 2LTRZFP Interacts Specifically to HIV-1 DNA without Off-Target Effects as Determined by Biolayer Interferometry

**DOI:** 10.3390/bios11030076

**Published:** 2021-03-08

**Authors:** Koollawat Chupradit, Weeraya Thongkum, On-anong Juntit, Kanokporn Sornsuwan, Chatchai Tayapiwatana

**Affiliations:** 1 Division of Clinical Immunology, Department of Medical Technology, Faculty of Associated Medical Sciences, Chiang Mai University, Chiang Mai 50200, Thailand; koollawat_c@cmu.ac.th (K.C.); weeraya.t@cmu.ac.th (W.T.); Onanong_jun@cmu.ac.th (O.-a.J.); kanokporn_sornsuwan@cmu.ac.th (K.S.); 2 Center of Biomolecular Therapy and Diagnostic, Faculty of Associated Medical Sciences, Chiang Mai University, Chiang Mai 50200, Thailand; 3 Center of Innovative Immunodiagnostic Development, Department of Medical Technology, Faculty of Associated Medical Sciences, Chiang Mai University, Chiang Mai 50200, Thailand; 4 Biomedical Technology Research Center, National Center for Genetic Engineering and Biotechnology, National Science and Technology Development Agency at the Faculty of Associated Medical Sciences, Chiang Mai University, Chiang Mai 50200, Thailand

**Keywords:** HIV-1, biolayer interferometry, zinc finger protein, 2LTRZFP

## Abstract

Protein and DNA interactions are crucial for many cellular processes. Biolayer Interferometry (BLI) is a label-free technology for determining kinetic biomolecular interactions with high accuracy results. In the present study, we determined the kinetic binding of a zinc finger scaffold, 2LTRZFP, which formerly constructed the interfering effect on HIV-1 integration process using BLI. The competitive Enzyme-linked immunosorbent assay (ELISA) was used to initially show the specific binding of 2LTRZFP. The percentages of inhibition were 62% and 22% in double-stranded 2LTR (ds2LTR) and irrelevant DNA (dsNeg), respectively. Consequently, the binding affinity of 2LTRZFP against ds2LTR target analyzed by BLI was 40 nM, which is stronger than the interaction of HIV-1 integrase (IN) enzyme to the 2LTR circle junction. Additionally, the 2LTRZFP did not interact with the genomic DNA extracted from SupT1 cell line. This result indicates that 2LTRZFP did not exhibit off-target effects against human genome. The knowledge obtained from this study supports the prospect of using 2LTRZFP in HIV-1 gene therapy.

## 1. Introduction

Regarding the drawbacks of antiretroviral therapy which is a current standard treatment for Human Immunodeficiency Virus-1 (HIV-1) infected patients, gene therapy is one of the approaches that has been developed as an alternative treatment towards HIV-1 infection. Several anti-HIV-1 molecules have been identified for use in HIV-1 gene therapy, including RNA-based methods [1,2], and protein-based method such as scaffold proteins and gene editing proteins, for example, designed ankyrin repeat protein, zinc finger nucleases (ZFNs) transcription activator-like effector nucleases (TALENs), and clustered regularly interspaced short palindromic repeats (CRISPR)/CRISPR-associated protein 9 (Cas9) [3,4,5,6,7].

A zinc finger protein (ZFP), namely 2LTRZFP, was formerly established by submitting the 18 base pairs of HIV-1 2LTR target sequence into the zinc finger tools software [8]. The designed 2LTRZFP consists of 6 contiguous ZFP motifs that have binding domains cover the range of HIV-1 2LTR target sequence. Its specific binding to the 2LTR junction of HIV-1 DNA deploys antiviral activity in the HIV-1 integration process. The study in human T cell lines and primary peripheral blood mononuclear cells (PBMCs) demonstrated that 2LTRZFP has promising effects in interfering HIV-1 integration and replication [8,9,10]. This protein can fold properly in cytoplasm and shows nuclear homing. However, the off-target effect of 2LTRZFP toward human genomic DNA has yet to be determined.

Several techniques have been applied for determining biomolecular interactions. Chromatin immunoprecipitation assays and sequencing (ChiP-Seq) assay is a technique that can detect DNA-protein interaction regardless of molecule labeling [11,12]. However, this technique is costly and requires experts to interpret the results. Isothermal titration calorimetry (ITC) is a physical technique used for studying small molecules by measuring the heat released or consumed during a binding event [13]. However, it is a low-throughput technique and it requires high sample consumption. Thus, ITC is not proper for screening of large compound library. Surface plasmon resonance (SPR) is a high sensitivity and label-free technique that utilizes interaction of light photons and free electrons on a metal interface. It measures changes in the refractive index at the sensor surface that is directly proportional to the mass of binding molecule. It can also determine kinetic binding interactions with high-throughput data analysis [14]. Nevertheless, this technique is suitable for high molecular weight molecules, and non-specific binding occasionally appears due to its high sensitivity [15].

The Bio-layer interferometry technology (BLI) is a label-free optical technique which examines the optical thickness changes at the biosensor surface after ligand binding. This technique is high-throughput and accessible with real-time kinetic binding detection [16,17]. From the previous study, BLI was demonstrated as a technique of choice for determining the kinetics and affinity constant of various biomolecular interactions. It provided the association rate (*k_a_*), the dissociation rate (*k_d_*), and equilibrium dissociation constant (*K_D_*) that were readily comparable to other biosensor platforms such as SPR [18]. As for a nucleic acid capture, Li and colleagues determined the binding affinity of the eukaryotic translation elongation factor 1A (eEF1A) protein to biotinylated 5’UTR, reverse transcriptase (RT), and luciferase RNAs. The results showed strong binding between eEF1A and 5’UTR RNA which indicates the specificity of 5’UTR RNA towards eEF1A monitored by BLI technique [19].

Nevertheless, most of the strategies mentioned earlier tend to detect on-target effects whether the molecules can specifically bind to each other. Off-target effects (i.e., interactions between molecule and unintended targets) are also necessary to distinguish since it can cause unexpected deleterious side effects. The yeast two-hybrid assay and affinity chromatography can monitor the binding of protein and ligand. However, the yeast two-hybrid assay cannot detect off-target effects while affinity chromatography is too sensitive, resulting in the unwanted non-specific bindings of the proteins [20].

Here we described the use of BLI technique for determining the specific binding of 2LTRZFP towards ds2LTR target. We also demonstrated the *K_D_* value under optimized experiment conditions whether the binding affinity of 2LTRZFP determined by BLI can correlate with the SPR technique that has been formerly reported [8]. Additionally, we verified the off-target effect of 2LTRZFP on human genomic DNA extracted from SupT1 cell line. The information obtained from this study can be used to elucidate various biomolecular interactions and develop anti-HIV-1 molecules for the future HIV-1 gene therapy treatment.

## 2. Materials and Methods

### 2.1. Recombinant 2LTRZFP-GFP and HIV-1 Integrase (IN) Enzyme Production and Purification

2LTRZFP-Green Fluorescent Protein (GFP) was prepared as described previously (8). Briefly, the plasmid vector pTriEx-4-2LTRZFP-GFP was transformed into *E. coli* strain Origami B (DE3). A single colony in a 5 mL Super broth pre-culture, supplemented with 100 μg/mL ampicillin, 12.5 μg/mL kanamycin, and 12.5 μg/mL tetracycline was cultured at 37 °C until reaching an absorbance at 600 nm of around 1.0–1.8. The pre-culture was diluted to 1:100 into 100 mL super broth medium with the same amount of antibiotics and supplemented with 100 μM Zn_2_SO_4_. The culture was incubated at 37 °C until reaching an absorbance around 1.0 at 600 nm. The bacterial culture was induced by 0.1 mM Isopropyl β-D-1-thiogalactopyranoside (IPTG) at 30 °C for 16–18 h. Bacteria culture was collected by centrifugation at 5000 rpm at 4 °C for 15 min. The bacterial pellet was resuspended in 6.25 mL of B-PER I Bacterial Protein Extraction Reagent (Pierce, Rockford, IL) and lysed by ultrasonication. The lysate was centrifuged at 15,000× *g* for 30 min at 4 °C. The supernatant was collected and filtered with microfiltration membranes (0.22-μm pore size). The clear solution containing His6-2LTRZFP-GFP was applied to HisTrap HP His tag protein purification columns (GE Healthcare Life Sciences, Marlborough, MA, USA) for protein purification. The purified protein was stored in 10–15% (*w/v*) final concentration of glycerol for long-term storage at −80 °C. The protein concentration was determined by using Pierce™ BCA Protein Assay Kit (Thermo Fisher Scientific, Waltham, MA, USA). The purified 2LTRZFP-GFP was analyzed by SDS-PAGE. Western blot analysis was performed with HRP conjugated anti-His tag monoclonal antibody (Biolegend, San Diego, CA, USA), using Pierce™ ECL Western Blotting Substrate (Thermo Fisher Scientific, Waltham, MA, USA).

HIV-1 integrase (IN) was expressed as a recombinant protein. The *E. coli* strain BL21 (DE3) containing plasmid pINSD. His.sol was cultured in super broth containing 100 μg/mL of ampicillin at 37 °C and 200 rpm until OD600 reaches 0.8. The 0.4 mM IPTG was added and incubated for 3 h. Bacterial cell pellets were resuspended in lysis buffer (including 1 M NaCl; 20 mM HEPES pH 7.5; 2 mM β-mercaptoethanol; 0.3 mg/mL lysozyme; 5 mM imidazole) and then incubated at 4 °C for 30 min and further lysed by ultrasonication. The lysate was centrifuged at 10,000 rpm for 30 min at 4 °C. The supernatant was collected and filtered through microfiltration membranes of 0.22 μM pore size. Protein was purified by HisTrap HP his-tagged protein purification columns. HIV-1 IN was then eluted with elution buffer (500 mM imidazole; 1 M NaCl; 2 mM β-mercaptoethanol; 25 mM HEPES; pH 7.5, and 10% W/V of glycerol). HIV-1 IN was performed dialysis in 0.5 M NaCl; 20 mM HEPES pH 7.5; 2 mM β-mercaptoethanol; 0.3 mM imidazole. The protein concentration was quantified by NanoDrop™ 2000/2000c Spectrophotometers (Thermo Fisher Scientific, Waltham, MA, USA). The purified HIV-1 IN enzyme was analyzed by SDS-PAGE. Western blot analysis was performed with HRP conjugated anti-His tag monoclonal antibody (Biolegend, San Diego, CA, USA), using Pierce™ ECL Western Blotting Substrate (Thermo Fisher Scientific, Waltham, MA, USA).

### 2.2. Double-Stranded 2LTR DNA Preparation

The 30-bp-length ds-2LTR DNA and irrelevant DNA (dsNeg) target were prepared for determining the binding activity of 2LTRZFP-GFP as described previously (8). A pair of specific ds-DNA (sense) was designed as follows: 5′-AAA TCT CTA GCA GTA CTG GAT GGG CTA ATT-3′ and a pair of nonspecific ds-DNA (sense) was also designed as follows 5′-TGA CAG TGC TAG CGT ATC ATC TAG TCG ACG-3′. The 100 μL of reaction mixture for annealing consisted of each single-stranded DNA (ss-DNA) and complementary strand in 50 mM NaCl. The mixture was firstly heated at 95 °C for 5 min and then cooled down to room temperature for 1 h. The 3’ end of the antisense strand of DNA was labeled with biotin.

### 2.3. Determination of the Binding Activity of 2LTRZFP-GFP towards Target DNA by Enzyme-Linked Immunosorbent Assay (ELISA)

To confirm the binding activity of 2LTRZFP-GFP to its target ds2LTR, the microtiter plate was coated with 10 μg/mL of avidin in coating buffer (1M NaHCO_3_, pH 9.3). The coated microtiter plate was left overnight in a moist chamber at 4 °C. Wells were washed three times with 0.05% Tween 20 in PBS and blocked the plate with blocking solution (2% BSA in PBS) 200 μL/well at RT in a moist chamber for 1 h to prevent non-specific binding. Next, washed three times, followed by adding 0.1 μM of 30-bp-length ds-2LTR-biotin in blocking solution. Incubated the reaction at RT in a moist chamber for 1 h. The 2LTRZFP-GFP at 2 μg/mL final concentration with or without 2 μM of ds-2LTR-biotin or dsNeg-biotin was added to the plate and incubated at RT for 1 h. The binding of 2LTRZFP-GFP to target DNA was revealed by incubation with an HRP-conjugated anti-His tag antibody at RT for 1 h, followed by addition of 50 μL of TMB microwell peroxidase substrate. The 6 N HCL was added to stop the reaction, and optical density (OD) was measured at 450 nm. The % inhibition was calculated as described below:

% inhibition = (OD_uninhibited_ − OD_inhibited_)/OD_uninhibited_ × 100

### 2.4. Binding Kinetic Analysis by Bio-Layer Interferometry

The 30-bp ds-DNA-biotin was prepared in zinc finger protein (ZFP) binding buffer (10 mM Tris-HCl, pH 7.5; 90 mM KCl; 1 mM MgCl2; 90 μM ZnSO4; 5 mM dithiothreitol; and 0.5 mM phenylmethylsulfonylfluoride). Next, the 0.5 μM of ds2LTR-biotin was immobilized on Streptavidin (SA) biosensors (FortéBio, Fremont, CA, USA) for 180 s, which was hydrated for 10 min before use. The ds-DNA-immobilized biosensor tips were placed into the zinc buffer for 30 s to set the binding baseline. The sensors were placed into the sample containing 2 μg/mL of 2LTRZFP-GFP for 200 s to determine the DNA-protein interaction in the presence or absence of 0.05 and 0.1 μM ds2LTR or dsNeg. Finally, the biosensor tips were placed in the zinc buffer for 100 s to observe dissociation signal. The association and dissociation will be measured using the BLItz biolayer interferometry (FortéBio, Fremont, CA, USA).

According to the inhibitory effect of 2LTRZFP-GFP on HIV-1 IN enzyme, the 0.5 μM of ds2LTR was immobilized on a SA biosensor tip and incubated for 120 s. After washing step, the ds2LTR immobilized biosensor was dipped into ZFP binding buffer containing 2 μg/mL of 2LTRZFP-GFP or GFP or only ZFP binding buffer for 120 s. The biosensor tip was dipped into 1:5 ratio of IN reaction buffer (25 mM HEPES pH 7.5, 10 mM DTT, 7.5 mM MgCl_2_, 0.05% Nonidet P-40, 30 mM NaCl, and 10 mM MgCl_2_) and ZFP binding buffer for 30 s to set baseline. The tip was then dipped into HIV-1 IN enzyme at 4 μM diluted in 1:5 ratio of IN reaction buffer and ZFP binding buffer and for 120 s to measure the HIV-1 IN binding activity. The biosensor tip was later dipped into 1:5 ratio buffer to observe the dissociation step.

To determine the off-target of 2LTRZFP-GFP to human chromosome, the genomic DNA from SupT1 cells was digested with *AluI* restriction enzyme. At the association step, the ds2LTR-immobilized biosensor tip was dipped into ZFP binding buffer containing 2 μg/mL of 2LTRZFP-GFP with or without 1 ng of digested SupT1 genomic DNA as an inhibitor. The association and dissociation were measured. The equilibrium dissociation constant (*K_D_*) was calculated from a non-linear local fit of the data by BLItz Pro 1.1 software.

### 2.5. Statistics

The paired Student’s *t*-test and one-way ANOVA were used for statistical analysis. Statistical significance was evaluated at ** *p* ≤ 0.01, *ns* > 0.05.

## 3. Results

### 3.1. Preparation of Recombinant Proteins

**Figure 1:**
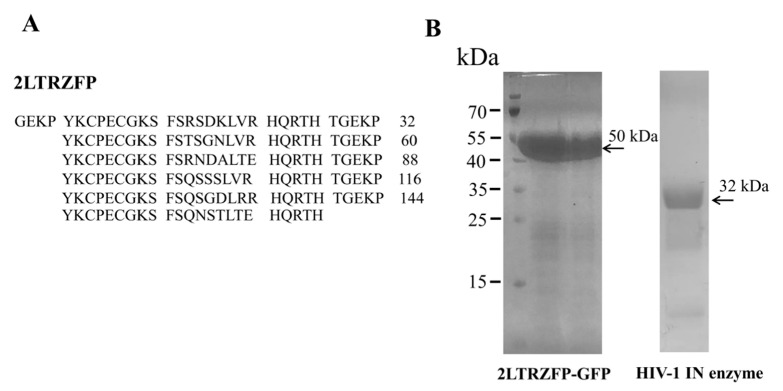
Amino acid sequence, production and purification of 2LTRZFP-GFP and HIV-1 IN enzyme. (**A**) Amino acid sequences of 2LTRZFP. (**B**) SDS-PAGE analysis of 2LTRZFP-GFP and HIV-1 IN purified by HisTrap HP His tag protein purification column. The molecular weights of recombinant 2LTRZFP-GFP and HIV-1 IN are 50 and 32 kDa, respectively.

To express the 2LTRZFP-GFP for determining its binding activity towards ds2LTR target. The recombinant 2LTRZFP-GFP was expressed in *E. coli* strain Origami B (DE3) while the HIV-1 IN was expressed in *E. coli* strain BL21 (DE3). The protein purification was achieved using HisTrap HP His tag protein purification column. The entire expression and purification processes were monitored by SDS-PAGE. The results showed the purity of fusion protein His6-2LTRZFP-GFP (~50 kDa) and HIV-1 IN (~32 kDa) after protein purification. This result suggested that the protein expression of 2LTRZFP-GFP and HIV-1 IN were successfully expressed and purified as shown in Figure 1.

### 3.2. Determination of 2LTRZFP-GFP Binding to Its Target DNA

**Figure 2:**
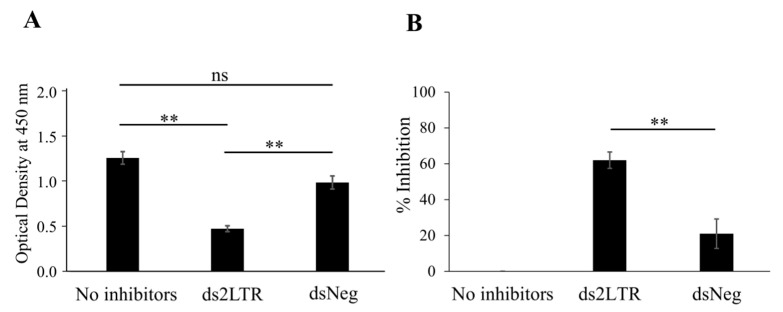
Competitive ELISA analysis for the specific binding of 2LTRZFP-GFP to ds2LTR DNA target. (**A**) The competitive reactions were reviewed by HRP-conjugated anti-His tag and measured optical density (OD) at 450 nm and (**B**) the signals obtained was converted into % inhibition. Data presented are from triplicate experiments (mean ± SD), analyzed using paired Student’s *t*-test. Statistical significance was evaluated at ** *p* ≤ 0.01, *ns* > 0.05.

To determine whether 2LTRZFP-GFP specifically binds to ds2LTR of HIV-1, the competitive enzyme-linked immunosorbent assay (ELISA) was performed. The results demonstrated that in the condition that did not contain DNA inhibitors or consisted of dsNeg as an inhibitor, the optical density (OD) values were 1.3 and 1.0, respectively. On the other hand, when having ds2LTR as an inhibitor, the OD dropped to 0.5 which is significantly lower than no inhibitors and dsNeg conditions (Figure 2A). Furthermore, the % inhibition of ds2LTR and dsNeg were 62% and 22%, respectively (Figure 2B). This suggested that 2LTRZFP-GFP can specifically bind to ds2LTR DNA target.

### 3.3. Binding Kinetics of 2LTRZFP-GFP Using BLItz

**Figure 3:**
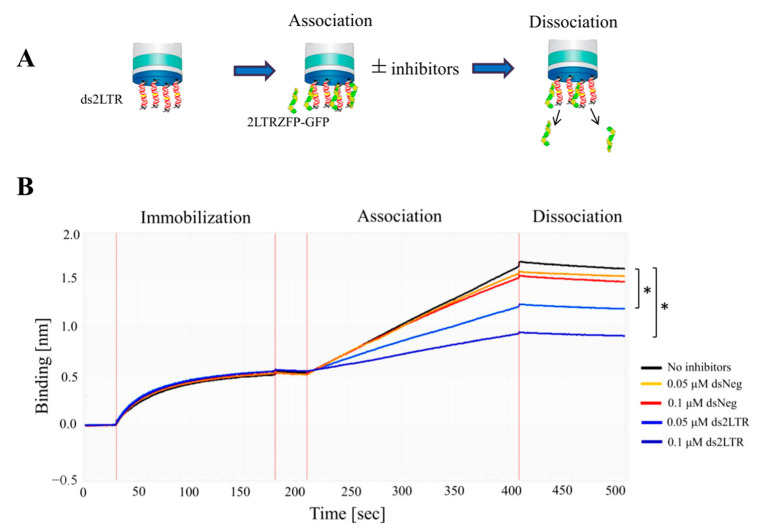
The binding kinetics of 2LTRZFP-GFP with its ds2LTR and dsNeg DNA target. (**A**) The schematic diagram of 2LTRZFP-GFP binding to target ds2LTR using BLItz^TM^ system. (**B**) Representative sensorgrams displaying the kinetics of the association and dissociation of 2LTRZFP-GFP towards biotinylated ds2LTR. The biotinylated 30-bp-length ds2LTR target was immobilized on streptavidin biosensor tips. After washing step, the ds2LTR immobilized biosensor tips was then dipped into the 2LTRZFP-GFP tube with or without inhibitors and the kinetic sensorgrams were obtained using a single channel ForteBio BLItz^TM^ instrument. Statistical significant differences of the association rate from triplicate experiments were analyzed by Student’s *t*-test at * *p* ≤ 0.05.

In this experiment, the binding kinetic of 2LTRZFP-GFP to DNA target was measured before testing with HIV-1 IN enzyme using the BLItz™ system. The biotinylated double strand 30-bp-length ds2LTR target at 0.5 μM was immobilized on streptavidin biosensor tips. After washing step, the ds2LTR immobilized biosensor tips was dipped into 2 μg/mL of 2LTRZFP-GFP tube and the kinetic sensorgrams were observed. The change in thickness (more or less) of the biological layer leads to the increase or decrease of binding signal that indicates the ongoing binding interaction. In Figure 3A, shown the schematic diagram of 2LTRZFP-GFP binding to ds2LTR using BLItz™ system. The Figure 3B demonstrated the results of the binding kinetic of 2LTRZFP-GFP. The results showed that in the condition that did not include inhibitors, the binding signal of 2LTRZFP-GFP with its ds2LTR DNA target was 1.2 nm (black line). On the other hand, in the conditions that contained ds2LTR at 0.05 and 0.1 μM as inhibitors showed significantly lower in binding signals which were 0.7 nm (light blue line) and 0.45 nm (blue line), respectively. The dsNeg control is a double-stranded DNA in which 2LTRZFP-GFP cannot bind to. At 0.05 and 0.1 μM of an inhibitor showed the binding signals at 1.1 nm (orange line) and 1.0 nm (red line) which were not significantly different compared to no-inhibition control. This study revealed that the specific binding between 2LTRZFP-GFP and ds2LTR can be determined using BLItz™ system.

### 3.4. Study of the Inhibitory Effect of 2LTRZFP-GFP on HIV-1 IN Enzyme

**Figure 4:**
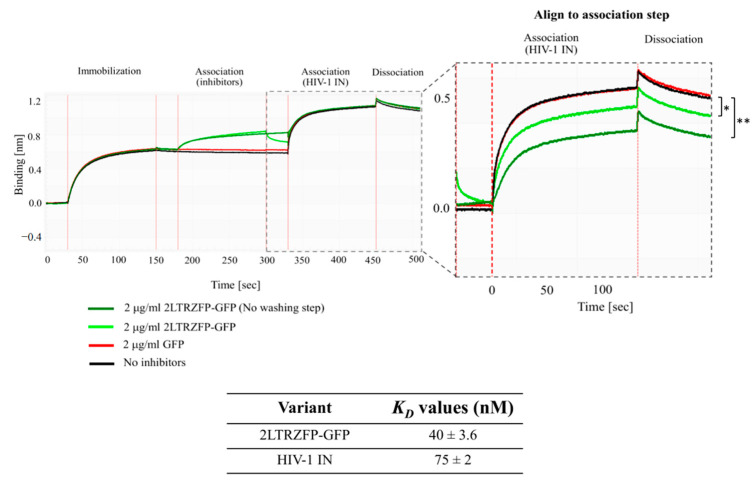
The inhibitory effect of 2LTRZFP-GFP on HIV-1 IN enzyme. Representative sensorgrams from triplicate experiments displaying the kinetics binding of HIV-1 IN towards biotinylated ds2LTR target in the condition that contained 2LTRZFP-GFP without washing step represented in dark green line. Whereas the bright green line was the competition with washing step. The GFP was used as an irrelevant control reperesented in red line. The *K_D_* values of 2LTRZFP-GFP and HIV-1 IN by BLItz^TM^ system were shown in the table. The significant differences in binding signals and KD values were analyzed by Student’s *t*-test; ** *p* ≤ 0.01 and * *p* ≤ 0.05.

From the previous experiments, the binding activities of 2LTRZFP-GFP with ds2LTR were determined. In this study, the inhibitory effect of 2LTRZFP-GFP on HIV-1 IN was determined using the BLItz technique. The 30-bp-length 2LTR DNA substrate was immobilized on the streptavidin biosensors. The DNA immobilized biosensor tip was then dipped into the tube containing 2LTRZFP-GFP. After washing out the excess proteins, the biosensors tip was dipped into HIV-1 IN tube to assess the binding activity of HIV-1 IN. In this study, purified Green Fluorescent Protein (GFP) was used as a negative control. The results in Figure 4 showed that in conditions of GFP (red line) and no inhibitors (black line), there were no binding signals of proteins observed at the step of inhibitors but when dipping biosensor tips in the HIV-1 IN, the binding signal went up to around 0.55 nm in both condition. This can be concluded that the binding signal generated after adding HIV-1 IN came from the actual binding of HIV-1 IN to the ds2LTR. These binding signals were baseline signals of HIV-1 IN binding to ds2LTR target. According to 2LTRZFP-GFP condition, when dipping the biosensor tips that already immobilized with ds2LTR in the 2LTRZFP-GFP, the binding signal of 2LTRZFP-GFP was observed (green line). After dipping into HIV-1 IN tube, the binding signal in the association step decreased from 0.55 nm to 0.47 nm when comparing to GFP and no inhibitor conditions. This could be the effect of 2LTRZFP-GFP that specifically bound to the ds2LTR and blocked the HIV-1 IN from binding to the DNA. However, when carefully observed the result of 2LTRZFP-GFP, after binding and washing the excess 2LTRZFP-GFP, the binding signal decreased a little at the dissociation step. This indicated that some 2LTRZFP-GFP was removed and left the ds2LTR target in the unbound form. Hence, the HIV-1 IN can still bind to the ds2LTR.

To confirm the inhibitory effect of 2LTRZFP-GFP on HIV-1 IN, the ds2LTR immobilized biosensor tips was dipped into 2LTRZFP-GFP tube and was not washed out the excess protein before testing with HIV-1 IN. The kinetic sensorgram showed the substantial decrease in binding signal of HIV-1 IN when comparing to no inhibitors or GFP from 0.55 nm to 0.37 nm (dark green line) as shown in Figure 4. This result suggested that 2LTRZFP-GFP can specifically bind to ds2LTR target leading to inhibiting the binding of HIV-1 IN enzyme using BLItz technique. The significant differences in binding kinetic were calculated using Student’s *t*-test.

To explain more about the reason why 2LTRZFP-GFP can interfere the binding of HIV-1 IN enzyme, the *K_D_* of 2LTRZFP-GFP and HIV-1 IN enzyme were determined. The results demonstrated that the *K_D_* values were 40 nM and 75 nM in 2LTRZFP-GFP and HIV-1 IN, respectively. It indicated that the inhibitory effect against HIV-1 IN was probably due to the binding affinity of 2LTRZFP-GFP that was a little higher than HIV-1 IN. Thus, the 2LTRZFP-GFP can inhibit the HIV-1 IN binding.

### 3.5. Off-Target Effect of 2LTRZFP-GFP to SupT1 Genomic DNA

**Figure 5:**
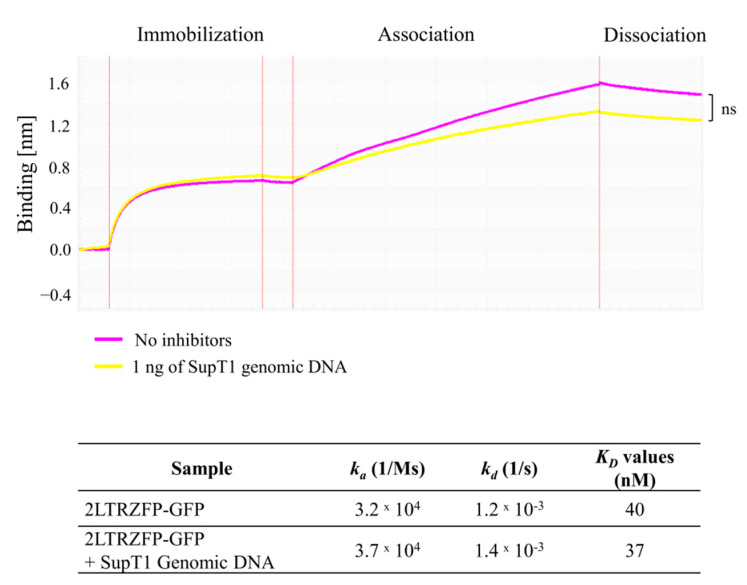
The off-target effects of 2LTRZFP-GFP towards SupT1 genomic DNA. The off-target effect of 2LTRZFP-GFP was determined by letting 2LTRZFP-GFP bind to SupT1 genomic DNA in the tube. The representative kinetic sensorgrams from triplicate experiments demonstrated the kinetic binding of 2LTRZFP-GFP in the condition with or without SupT1 genomic DNA. Statistical significance was evaluated by Student’s *t*-test; ns, not significant.

It is necessary to determine the off-target effect of 2LTRZFP-GFP since this protein specifically binds to 2LTR circle junction of HIV-1 DNA. To prove that the 2LTRZFP-GFP does not have off-target effect towards human genome, genomic DNA extracted from SupT1 cells line was firstly digested with *AluI* restriction enzyme to make it smaller and tested the binding with 2LTRZFP-GFP. The results in Figure 5 demonstrated the binding signal around 1.0 nm in the condition that did not contain genomic DNA from SupT1. Likewise, 1 ng of SupT1 genomic DNA did not inhibit the binding of 2LTRZFP-GFP to ds2LTR-immobilized biosensor with the binding signal at 0.7 nm. Additionally, the *K_D_* values in both conditions were not significantly different determined by Student’s *t*-test which is indicating that 2LTRZFP-GFP did not exhibit off-target effects against SupT1 genomic DNA.

## 4. Discussion

Currently, the therapeutic proteins have become a powerful treatment in various diseases [21]. Most of them are now in clinical trials, for example, cancer therapy, immune disorders, and infections [22]. HIV-1 infection is one of the infectious diseases that is very challenging to find the cure without antiretroviral drug administration. Various gene-based therapies have been developed to function in different steps of HIV-1 life cycle. Designed scaffold proteins display high durability and stability during the expression [23]. In addition, the binding sites of scaffold proteins can be modified to target specifically to HIV-1 DNA or proteins resulting in blocking HIV-1 infection. However, the key to success of using protein-based therapy is to find an efficient and safe molecule for applying in HIV-1 infected patients.

In this present study, we determined the specific interaction of 2LTRZFP-GFP to its target ds2LTR HIV-1 DNA. The % inhibition demonstrated that 2LTRZFP-GFP specifically bound to ds2LTR which plays an important role HIV-1 integration process. Moreover, we showed the applicability of biolayer interferometry (BLI) to detect a scaffold protein binding to DNA. The BLI signals that we obtained during the capture of 2LTRZFP to ds2LTR are mostly the results of direct binding leading to an increase in thickness on the biosensor surface. Furthermore, the function of 2LTRZFP has been evaluated whether it has an ability to inhibit HIV-1 IN binding. The results showed that when 2LTRZFP-GFP bound to the ds2LTR target, it can efficiently inhibit the binding of HIV-1 IN enzyme which was due to the higher binding affinity of 2LTRZFP-GFP comparing to HIV-1 IN. The data obtained from this study suggested that BLItz can detect the kinetic inhibitory effect of 2LTRZFP-GFP towards HIV-1 IN. Several studies used BLItz technique for research in many aspects, for example vaccine development, epitope mapping, antibody design, virus screening [24]. Interestingly, in 2016 Verzijl D. and colleagues established a cell-based BLI for the first time to determine the real-time signal transduction in living cells [25]. Recently, Muller-Esparza and colleagues utilized BLItz to measure CRISPR-Cas9 complexes binding to biotinylated oligonucletides. The results showed the binding kinetic information can be corroborated with MicroScale Thermophoresis (MST) technique [26]. This indicated that BLI is a reliable technique for determining protein and nucleic interaction.

Lately, several anti-HIV-1 molecules and gene editing techniques have been developed to target or inhibit HIV-1 infection. However, off-target effects can be unavoidable when DNA binding proteins or gene-editing molecules bind at closely matched sequences in the genome [27]. There are many strategies for detecting on- and off-target effects such as computational prediction, mass spectrometry-based proteomics, and ChiP-Seq assay [20,28,29]. The limitations from those techniques are low-throughput, time-consuming and expensive. Even though CRISPR-Cas9 system is a powerful method for gene editing, off-target mutagenesis is still a problem [30,31]. In 2020, Coelho and colleagues have developed CRISPR GUARD strategy to reduce Cas9 off-target effects without decreasing on-target editing efficiency. The results from BLI demonstrated that the majority of off-targets were protected by CRISPR GUARD molecule [32]. In our study, the off-target effect of 2LTRZFP-GFP was determined to observe whether 2LTRZFP-GFP can exhibit non-specific binding towards human Chromosome using BLI system. The sensorgram and calculated *K_D_* values showed no significant differences between the condition that consisted of digested SupT1 genomic DNA or no inhibitors. It indicated that 2LTRZFP-GFP binds specifically to ds2LTR target and does not display off-target effects to human chromosome.

Altogether, the data obtained from this study suggested that 2LTRZFP is a safe anti-HIV-1 molecule for HIV-1 gene therapy application. Moreover, BLI is a fast and powerful technology and could be useful for developing a new method for monitoring molecule-ligand interactions, off-target effects, HIV-1 resistance strains, or drug screening in the future.

## Data Availability

Not applicable.
